# *Vibrio* Proteases for Biomedical Applications: Modulating the Proteolytic Secretome of *V. alginolyticus* and *V. parahaemolyticus* for Improved Enzymes Production

**DOI:** 10.3390/microorganisms7100387

**Published:** 2019-09-24

**Authors:** Monica Salamone, Aldo Nicosia, Giulio Ghersi, Marcello Tagliavia

**Affiliations:** 1 Abiel s.r.l, via del Mare 3, 91021 Campobello di Mazara (TP), Italy; m.salamone@abielbiotech.com (M.S.); giulio.ghersi@unipa.it (G.G.); 2 IRIB-CNR (Institute for Biomedical Research and Innovation-National Research Council), Via Ugo La Malfa 153, 90146 Palermo, Italy; aldo.nicosia@cnr.it; 3 Department of Biological, Chemical and Pharmaceutical Sciences and Technologies (STEBICEF), University of Palermo, Viale delle Scienze, Ed. 16, 90128 Palermo, Italy

**Keywords:** *V. alginolyticus*, *V. parahaemolyticus*, proteolytic secretome, collagenase, proteases production

## Abstract

Proteolytic enzymes are of great interest for biotechnological purposes, and their large-scale production, as well as the discovery of strains producing new molecules, is a relevant issue. Collagenases are employed for biomedical and pharmaceutical purposes. The high specificity of collagenase-based preparations toward the substrate strongly relies on the enzyme purity. However, the overall activity may depend on the cooperation with other proteases, the presence of which may be essential for the overall enzymatic activity, but potentially harmful for cells and tissues. Vibrios produce some of the most promising bacterial proteases (including collagenases), and their exo-proteome includes several enzymes with different substrate specificities, the production and relative abundances of which strongly depend on growth conditions. We evaluated the effects of different media compositions on the proteolytic exo-proteome of *Vibrio alginolyticus* and its closely relative *Vibrio parahaemolyticus*, in order to improve the overall proteases production, as well as the yield of the desired enzymes subset. Substantial biological responses were achieved with all media, which allowed defining culture conditions for targeted improvement of selected enzyme classes, besides giving insights in possible regulatory mechanisms. In particular, we focused our efforts on collagenases production, because of the growing biotechnological interest due to their pharmaceutical/biomedical applications.

## 1. Introduction

Proteolytic enzymes, including those of microbial origin, are employed in several fields, ranging from food manufacturing/processing, to the enzymatic treatment of various wastes for the production of highly valuable by-products, as well as biomedical-pharmaceutical applications. In this scenario, enzymes showing high tolerance to various harsh or particular conditions (pH, temperature, salt content, etc.) are required for industrial and biomedical applications. Such features are expected for enzymes from bacteria thriving in extreme/unique environments, where studies focused on bacterial assemblage and metabolism [1,2] and references therein, would deserve further efforts aiming to the identification of possibly attracting enzymes. However, such abilities are found in many marine enzymes (even from not extremophiles bacteria), as well, which are often considered among the most valuable biocatalists for a wide range of uses, including industrial and biomedical applications [3,4,5,6].

Members of genus *Vibrio* are among the most promising, well recognized as proteases-producing bacteria, worthy to be further considered and explored as source of enzymes and other biomolecules [7]. 

Many proteolytic enzymes are secreted, and their production is directed to the transformation of high molecular weight polypeptides into shorter chains, for an easier uptake and utilization, but they may act as virulence factors in pathogenic bacteria. Such enzymes are of high interest for biotechnological applications [7,8,9]; in particular, proteases from marine organisms are particularly attractive and promising for biomedical and experimental sciences such as tissue dissociation and cell isolation for transplantation/cell-therapy purposes [9,10]. 

Among proteases from *Vibrio spp.* (one of the main proteases-producing bacteria), collagenases have attracted interest for a long time, and some of them are routinely used for biomedical and pharmaceutical uses. 

*Vibrio* collagenases are zinc metalloproteinases classified into subfamily M9A in the MEROPS database [11]. Collagenases belong to a family of zinc-dependent metalloproteinases that degrade collagen substrates, and they are naturally produced by most animals and by many bacteria. 

The main advantage of bacterial collagenases relies on their ability to carry out the selective removal of necrotic tissue [12,13] with only minor involvement of the neighboring healthy tissues [14,15]. Therefore, they have been successfully applied in the treatment of many pathological conditions [16] including third-degree burns [17,18], ulcers [19,20,21], ischemic arterial ulcers [22,23] and Dupuytren’s disease [24]. More recently, collagenases have been approved by both the FDA and the European Union to be used as nonsurgical treatment for Peyronie’s disease. Moreover, collagenases have been used in laboratory procedures involving tissues dissociation and cells isolation [9,25,26,27,28,29].

Overall, these features and range of application make collagenases precious supporters for a variety of situations.

The secreted collagenase from *V. alginolyticus* is being successfully applied for pharmaceutical uses [30] and references therein] that exhibits limited non-specific proteolytic activity, which reduces side effects due to digestion of healthy tissues [31]. Therefore, such enzyme, which is industrially produced by autologous expression, has proven to be useful and effective as debriding agent for medical uses (i.e., ointments and other devices).

*Vibrio alginolyticus* is a halophilic Gram-negative γ-proteobacterium mainly occurring in warm marine and estuarine environments. It is a pathogen, sometimes opportunistic, for both humans (causing mainly wound infections) and sea animals [32,33,34,35,36,37,38,39]. 

The pathogenesis mechanism of *V. alginolyticus* has not been fully understood, but several virulence factors including, among others, secreted proteases, collagenases and siderophores, have been described [40,41,42,43,44,45,46], which supports the idea that, similarly to other pathogenic bacteria (like *V. vulnificus*), such a microorganism could take advantage of proteases, as well as iron availability, for effective host attack and bacterial dissemination through digestion of the tissues’ protein components [45,46,47,48,49]. 

*V. parahaemolyticus* is very closely related to *V. alginolyticus* and, similarly to the latter, in recent years is becoming the leading foodborne pathogen responsible for food poisoning and acute gastroenteritis [50,51,52,53,54,55,56]; it also causes wound-infection through exposure of a new wound to contaminated seawater or estuarine water [10]. In *V. parahaemolyticus*, the collagenase VppC has been also described [57] 

The production of collagenase in *V. alginolyticus* is known to be regulated by multiple factors, including collagen and its high molecular weight fragments, as well as peptides, quorum sensing temperature and oxygen [58,59,60,61,62], whereas the factors regulating collagenases production in *V. parahaemolyticus* are not well established. 

Besides collagenases, the secreted proteolytic proteome of both *Vibrio* strains includes various enzymes with possible biotechnological applications.

With the aim of identifying cultural conditions that could improve either the overall proteases production or that of specific enzymes, namely collagenases and/or other proteases, we tested ten different culture media, so as to seek for the best cultural conditions to be set for maximum yield of target enzymes.

The rationale was based, for many assayed conditions, on the knowledge of the factors known as supportive for collagenase induction, as well as on our preliminary observations in closely related bacteria for the production of various secreted proteases. We reasoned that both *V. alginolyticus* and *V. parahaemolyticus* are pathogenic bacteria, which benefit of secreted proteases for host invasion, and that the production of such enzymes might be expected to be enhanced by factors mimicking the host environment. Moreover, we wondered if the production of secreted proteases, including collagenases, could be enhanced by factors well known to be involved in the virulence of many pathogens. It is worth noting that published studies are focused on collagenase expression (the main enzyme of interest due to its biomedical/pharmaceutical uses), while little or no information is available about the overall proteolytic proteome modulation.

## 2. Materials and Methods

### 2.1. Microbiological Methods

#### 2.1.1. Culture Media and Conditions

Marine Broth (MB, Laboratorios CONDA, Spain) and Marine Agar (MB containing 1.5% Agar) were used for strains maintenance and routine cultivation, as well for intermediate inoculum. 

MB was used as base medium for subsequent modifications, consisting of dilution 1:5 in sterile seawater (for MB 0.2×), or in the addition of 1% Gelatine, Type B, from bovine skin (SigmaAldrich, Milan, Italy) (MB+Gel), Casaminoacids 5 g/L (Sigma) (MB+AA), Yeast Extract (Conda, Madrid, Spain) 9 g/L (MB+YE), Peptone (Sigma) 9 g/L (MB+Pept), Glucose 4 g/L (MB+Glc), 0.5% Glycerol (MB+Gly), Meat Extract (Sigma) 10 g/L (MB+ME), FeCl_2_.EDTA 10 µM (MB+Fe).

Type strains were inoculated from single colonies, grown onto Marine Agar, in MB and let to grow overnight at 28 °C in an orbital shaker. The resulting liquid cultures were used to inoculate the aforementioned media by dilution of 1:1000.

Cultures were grown for 18 h at 28 °C in an orbital shaker for proper aeration (220 rpm), then OD_600_ was measured for each culture and the cells harvested by centrifugation (10 min at 8000× *g* at 4 °C) after chilling on ice for 10 min. 

The resulting cell-free medium was used for biochemical assays. Data refer to at least three independent replicates. Error bars in the graphs represent 1 standard deviation.

#### 2.1.2. Type Strains

*Vibrio* type strains were from DSMZ (Germany), and in particular *V. parahaemolyticus* (DSM10027) and *V. alginolyticus* (DSM2171).

### 2.2. SDS Electrophoresis and Zymography

Sodium dodecyl sulphate-polyacrylamide gel electrophoresis (SDS-PAGE) was carried out as described by Laemmly. After electrophoresis, the gels were stained with 0.25% Coomassie Brilliant Blue G-250. The molecular weight of the enzyme was estimated using a molecular weight markers. Zymography was performed on substrate supplemented-PAGE. After electrophoresis, gelatine zymographies were incubated for 24 h at 37 °C in two developing buffers: Activator buffer containing 2 mmol/L CaCl_2_, Tris-HCl buffer (50 mmol/L; pH 7.4), containing 1.5% Triton X-100 and 0.02% Na Azide and Inhibitor buffer Tris-HCl buffer (50 mmol/L; pH 7.4), containing 1.5% Triton X-100 and 0.02% Na Azide plus 2 mmol/L EDTA to inhibit any gelatinase activity. After incubation, gels were stained using Coomassie Brilliant Blue G-250.

### 2.3. Substrate Specific Activity Determination

Total protease activity was measured by azocasein assay according to the protocol [63] with some modification. The principle of this assay is the hydrolysis of azocasein by proteases resulting in release of azo-molecule with absorption at 450 nm. The assay was carried out by incubating 50 µL of supernatant with 200 μL of 1% azocasein in 0.2 M Glycine-NaOH (pH 7.5) at 37 °C for 4 h. The reaction was terminated by the addition of 300 μL of 5% trichloroacetic acid. The assay mixture was centrifuged at 10,000× *g* for 10 min, then an equal volume of 1.0 N NaOH was added to the supernatant and absorbance was measured at 450 nm. Calibration curve was made according to Reference [64].

The collagenolytic activity was measured following the procedure described in Reference [65] using a modification of the collagen digestion method, in which the enzymes were incubated for 5 h with native bovine Achilles tendon collagen (Sigma-Aldrich, St. Louis, MO, USA) at 37 °C. The collagen digestion was determined using the colorimetric ninhydrin process [66]. The amino acids released are expressed as micromoles leucine per milligram dry weight of enzyme. One unit equals one micromole of leucine equivalents released from collagen in 5 h at 37 °C, pH 7.5, under the specified conditions [8]. Enzymatic activities were calculated considering the values obtained from each sample both in presence and in absence of substrate (the latter was subtracted, so as to avoid any contribution of culture medium components).

### 2.4. Sequence and Structural Analyses of Collagenases

Functional sites and domains in the predicted amino acid sequences were predicted using the InterProScan software [67] the Simple Modular Architecture Research Tool (SMART) program, the Pfam database [68] and the PROSITE program [69] MSA were constructed using T-Coffee (Tree-based Consistency Objective Function For alignment Evaluation). The 3D structures were reconstructed by homology modelling via the Protein Homology/analogY Recognition Engine 2.0 (Phyre 2) software [70] using the intensive modelling model. Candidate structures for homology modelling were selected according to pairwise alignment. At least two different structures were used as a template for each generated structure as reported elsewhere [71,72,73,74,75] and homology models were built for all the sets of proteins. 

Additionally, the evolutionary variability of amino acids onto the reconstructed structures were computed using ConSurf webserver [76] and rendered using the UCSF Chimera package.

## 3. Results and Discussions

### 3.1. Culture Media and Growth Performances

Marine Broth (MB) was chosen as standard/reference medium, because of its well-recognized effectiveness in supporting the growth of marine vibrios. Moreover, its composition makes it suitable for large scale production, where its synthetic salty component may be replaced by seawater. Finally, its recipes include peptone, which has been reported as collagenase inducer in *V. alginolyticus* [59]. Therefore, we decided to test various modification of such a culture medium, seeking for compositions able to enhance enzymes production. Moreover, a diluted MB was tested, as well.

Both *V. parahaemolyticus* and *V. alginolyticus* were challenged with all cultural media, and the growth performances, as well as the production of secreted proteases were compared. 

**Figure 1:**
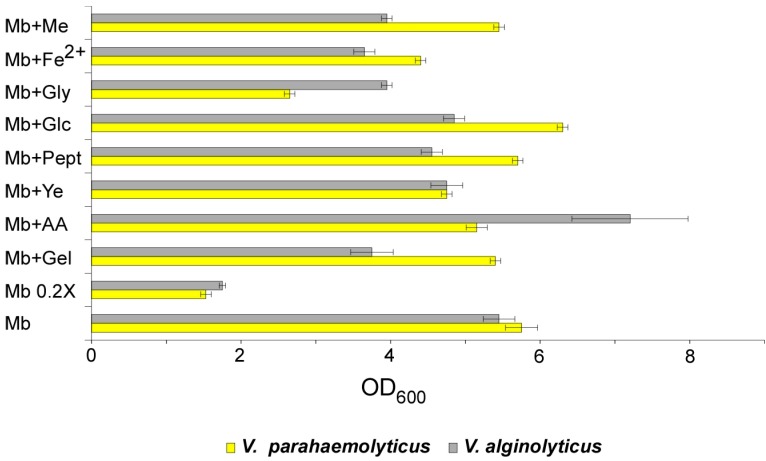
Growth performances of *V. parahaemolyticus* and *V. alginolyticus* in different culture media. *V. parahaemolyticus* and *V. alginolyticus* were grown overnight in the specified liquid media, and the cells density was measured spectrophotometrically as OD_600_. Mb: Marine broth; Gel: Gelatine; AA: Casaminoacids; Ye: Yeast extract; Pept: Peptone; Glc: Glucose; Gly: Glycerol; Fe: FeCl_2_.EDTA; Me: Meat extract.

Almost all formulations supported a good bacterial growth, with a nearly overlapping trend (except for some differences) for both *Vibrio* strains (see Figure 1).

In particular, the 0.2× dilution of MB resulted in severely reduced growth, as expected; the addition of gelatine (from mammalian skin), as well as of glycerol, iron and meat extract, resulted in some growth reduction for *V. alginolyticus*; minor growth reduction was recorded in glucose, peptone or yeast extract supplemented media. Only the supplementation with casamino acids (mostly consisting of free amino acids from casein hydrolysate) resulted in a growth enhancement.

*V. parahaemolyticus* responded in a similar way to various conditions, with an overall growth reduction compared to MB; glycerol had stronger effects, compared to *V. alginolyticus*, while a slight growth improvement was observed in presence of glucose.

As general rule, the growth and metabolic efficiency of non-oligotrophic bacteria is positively modulated by nutrients concentration and availability, although growth efficiency and enzyme production are not necessarily related (see Section 3.4). In particular, carbon/nitrogen sources as protein and or cell hydrolysates (i.e., peptones, casaminoacids, yeast extract, meat extract, etc.) are well known for their stimulating activity on bacterial growth. 

Besides their ability to increase the overall nitrogen/carbon sources concentration, they greatly differ not only in compositions, but also for the ease of nutrients uptake which, in turn, results in a different “available nutrients concentration” the cells are exposed to over time.

Peptone and casamino-acids are pure source of amino acids, consisting of peptides from protein enzymatic digestion and casein acid hydrolysate (i.e., mainly free amino acids and very short peptides) respectively, the latter being fully available immediately. Whereas, yeast extract (from yeast autolysate) and meat extract contain, besides peptides and amino acids, a complex mixture of low molecular weight nutrients ranging from nucleotides to trace elements (including iron, especially for meat extract). In fact, meat extract is manufactured from meat (with low fat and sinew content) and can be considered as complementing the nutritive properties of peptone by contributing minerals, phosphates, energy sources and those essential factors missing in peptone. 

Since the possibility cannot be excluded that some of these—even minor—components could mimic the infected tissues microenvironment (which might stimulate virulence, as well as productions of secreted factors including degradative enzymes, even if an opposite effect might be hypothesized as hydrolysis products, as well), possible positive effects not only on the growth, but first all on enzymes production may be reasonably expected.

Most of the aforementioned recipes are widely used, even in high concentrations, in several fermentation culture media, including those recommended for production of recombinant proteins, where extra nutrients and energy sources (i.e., glucose and glycerol) are recognized as beneficial in supporting the growth and the protein synthesis overload. However, most of such knowledge has arisen from production of recombinant proteins, which is usually not affected by other global regulatory mechanisms. In contrast, the response to cultural conditions at the level of gene expression and proteins production in autologous systems is not so obvious or predictable, and it was the focus of the following experiments.

### 3.2. Collagenase Production

Collagenases are probably the most valuable enzymes occurring in *Vibrio* secretome. In order to assess their actual production, the overall collagenolytic activity was measured in supernatants from each culture.

**Figure 2:**
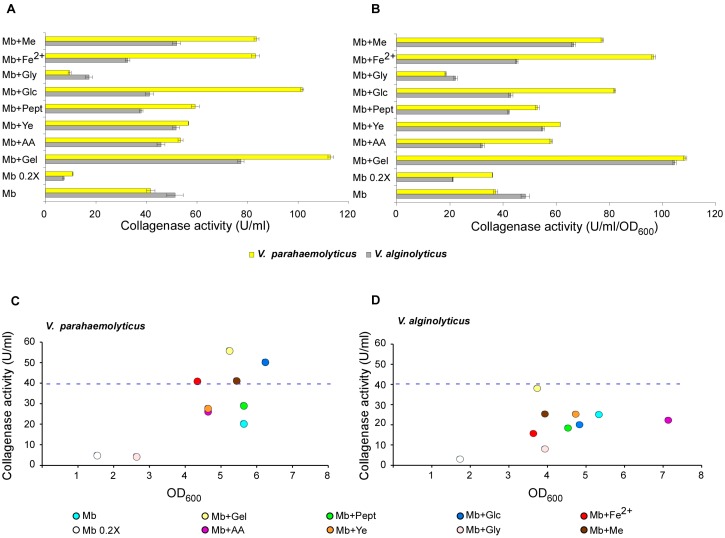
Collagenase production from *V. parahaemolyticus* and *V. alginolyticus* in different culture media. The collagenolytic activity was measured using bovine insoluble collagen as substrate. Collagen was incubated with supernatants from liquid cultures of *V. parahaemolyticus* and *V. alginolyticus*, grown overnight in the specified liquid media. (**A**) Collagenase activity measured by colorimetric test. (**B**) Collagenolytic activity normalized with cell density. (**C**,**D**) Trend of collagenolytic activity with cell density in *V. parahaemolyticus* (**C**) and *V. alginolyticus* (**D**).

In particular, the degradative activity was assessed using insoluble collagen from bovine tendon as substrate, instead of a synthetic [77] one. In fact, while the latter is recognized to dramatically increase the specificity for collagenases, it does not provide information about the enzyme activity toward natural substrates, because it selects for enzymes able to recognize the specific amino-acidic sequence in denaturated collagen. In contrast, measuring the digestion of a native substrate is the test of choice to assess the actual effectiveness of proteases in digesting native collagen, being the latter a quite peculiar substrate, difficult to be hydrolysed because of its structure and accessibility of cleavage sites [78]. Results showed a variety of responses to different culture conditions, and significant differences between the two strains (see Figure 2A).

In particular, *V. alginolyticus* collagenase production was strongly reduced in low nutrients conditions (MB 0.2×), in presence of glycerol and, at lower extent, in the iron-supplemented medium; little variations were observed in other media, except for the gelatine-based one, which was confirmed [58] to improve the collagenolytic activity.

Instead, a very different behaviour was observed in *V. parahaemolyticus,* where an overall improvement of collagenase production was observed in all culture media, except for MB 0.2× and MB supplemented with glycerol (where, similarly to *V. alginolyticus,* a clear reduction of collagenase activity occurred). Such enhancements became dramatic, ranging from two to three folds, in presence of either gelatine or glucose, as well as in media supplemented with either iron or meat extract.

Further interesting insights into the physiological response to the tested conditions could be gained considering the production normalized per cell density (Figure 2B–D). The normalization of collagenase activity with cell concentration clearly shows that the enzyme production in *V. alginolyticus* is hampered by all medium modification, except for supplementation with meat extract (which resulted in about 40% induction) and, negligibly, yeast extract. Instead, gelatine proven to be the only strong inducer of collagenase production, and had the same positive effects on *V. parahaemolyticus*, as well.

The latter species showed a very different response pattern (compared to *V. alginolyticus*), nearly overlapping with that of the overall measured activity values in the medium, where most supplementations showed positive effects; however, the strongest collagenase induction was achieved in presence of either gelatine or iron (in contrast, the latter was ineffective in *V. alginolyticus*).

Results obtained in *V. parahaemolyticus* showed a high responsiveness of this bacterium to various supplementations in terms of collagenase production, which were more strongly enhanced by gelatine, meat extract, glucose and iron. The possibility that such compounds could affect the enzymatic activity, either directly (through enzyme modulation) or indirectly (by stimulating the production of modulator factors) is unlikely, as none of such mechanisms have been described for *Vibrio* enzymes, to date; this prompted us to consider any enzymatic activity variation as ascribable to differences in enzyme production.

The knowledge that culture media containing animal derivatives (like gelatine or meat extract) raises concerns for human health when products are intended for biomedical/human uses, prompted us to investigate conditions that could further improve collagenase production without the addition of gelatine as inducer.

In particular, we wondered if the combination of iron and glucose could exert additive effects in *V. parahaemolyticus* collagenase induction, compared with their use as single supplement.

**Figure 3:**
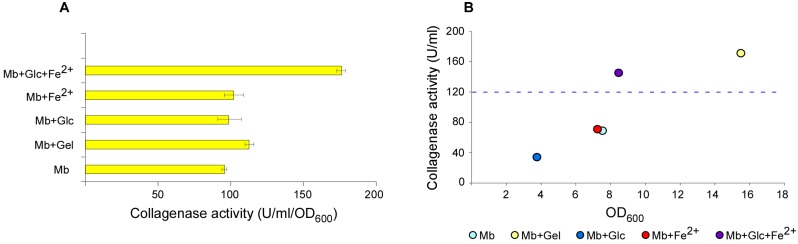
Collagenase production from *V. parahaemolyticus* with combinations of non-animal inducers. *V. parahaemolyticus* was grown overnight in the specified liquid media and collagenase activity measured by colorimetric test, using bovine insoluble collagen as substrate. Collagenolytic activity normalized with cell density (**A**) and trend of collagenolytic activity with cell density (**B**).

Actually, combining the two inducers exerted a strong effect in terms of production per cell (induction), whose increase exceeded about 70% and 80% compared with gelatine and MB, respectively (Figure 3A). However, the overall yield could not exceed that obtained with gelatine alone, because of the much higher cell growth obtained in the latter condition. (Figure 3B).

Further efforts, consisting of various combination of media composition with cultural strategies (including fed-batch), could reasonably result in further improvements in the overall enzyme yield and a best “matching” between overall and “per cell” collagenase production.

It is noteworthy that the addition of glycerol to the growth media, even in presence of inducers, seems to “turn off” the enzyme production. Such response was rather unexpected and would deserve further investigations aiming to explain how this small, easily metabolizable molecule, could exert such a strong effect, presumably at transcriptional level and/or, less likely, affecting the secretion. Moreover, the possibility that glycerol could induce an overall reprogramming at the gene expression level (i.e., affecting processes ranging from transcription to RNA metabolism, to translation and downstream steps), possibly responsible for the observed impaired cell growth (see Figure 1), should be considered and investigated.

In this regard, it must be noted that the expression of *V. alginolyticus* collagenase undergoes some long-term regulation, as its production shows some rifampicin-insensitivity [59], which let us hypothesize an extremely long mRNA half-life (of at least 60 min, based on data reported in Reference [59]), rather than a rifampicin-resistant transcription. If confirmed, this implies that any modulation at transcriptional level exerts its effects over a long time (this might be exploited for enzyme production when using metabolizable inducers, the effects of which have to be expected to extend for a long time after their depletion). 

Accordingly with the aforementioned possible mechanisms, the simulation of the *V. alginolyticus* colA 5’-UTR mRNA folding showed similarities with *E. coli* ompA 5’-UTR [79,80], one of the best characterized models for bacterial long-lived mRNAs, the stability of which has been reported to be affected, among other factors, by glucose [81].

Taken together, such elements encourage further investigations aiming to better understand such mechanisms. 

### 3.3. Structural Analysis of Collagenases from V. parahaemolyticus and V. alginolyticus

Keeping in mind the recognized high closeness of the two *Vibrio* species, in order to get insights into the overall similarity and structural relationships between their collagenases, bioinformatic analyses were performed. In fact, depending on the degree of relativeness between enzymes already approved for practical uses and others, overlapping features might be hypothesized, thus supporting further efforts aiming to evaluate the possibility of actual use of new enzymes (namely, collagenase from *V. parahaemolyticus*) for similar applications.

**Figure 4:**
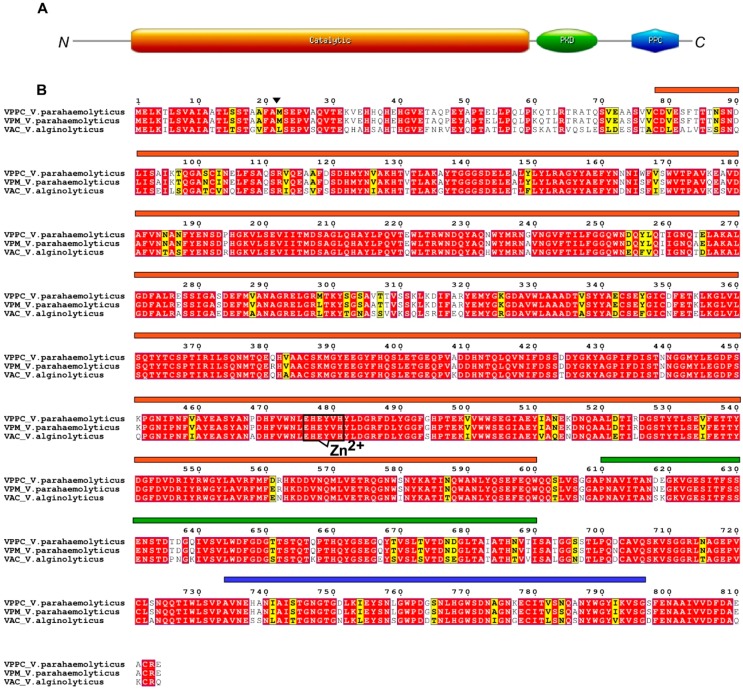
Schematic diagram of M9A class III collagenases from *V. parahaemolyticus* and *V. alginolyticus*. (**A**) Collagenases possess a N-terminal signal peptide and a proteolytic activation site marked by an arrow. The catalytic, the PKD-like and PPC domains are showed in orange, green and blue, respectively. (**B**) Sequence alignment of collagenases from *V. parahaemolyticus* and *V. alginolyticus*. Amino acids forming the zinc-binding motif are shown. Similar residues are written in black bold characters and boxed in yellow, whereas conserved residues are in white bold characters and boxed in red. The alignment was performed with T-Coffee. The sequence numbering on the top refers to the alignment.

*V*. *alginolyticus* produces a collagenase secreted as a 82 kDa protein (VAC) which show the typical domain organization of class III enzyme of M9A subfamily (Figure 4A,B). It is synthesised as inactive precursor with a putative tripartite N-terminal signal peptide (residues 1–21), required for translocation across the inner membrane. VAC possesses a cleavage site for proteolytic activation located between position 21 and 22 and a catalytic domain (residues 78–600) including a canonical zinc-binding motif (H^477^EYVH^481^). A PKD (Polycystic Kidney Disease) domain (residues 609–697), consisting of a beta-sandwich of seven or more strands in two sheets with a Greek-key topology, was also found together with a prepeptidase (PPC) domain (residues 735–796) at the C-terminal of VAC.

*V. parahaemolyticus* produces two 90 kDa proteins belonging to class III collagenases, VppC and VPM, which are synthetized as inactive precursors with the canonical tripartite signal peptide for translocation (residues 1–21) followed by a proteolytic activating cleavage site located between residues Ala21 and Met22. Similarly to the homolog from *V*. *alginolyticus*, both VppC and VPM show the two domains organization with the catalytic domains (residues 78–600 in each proteins), harbouring the motif H^477^EYVH^481^ responsible for Zn^2+^ coordination, followed by the PKD (residues 609–697) and the PPC domains at their corresponding carboxyl terminus.

Additionally, *V. parahaemolyticus* also produce class II protease named PrtVp possessing the catalytic domain with a zinc-binding motif (HEYTH) lacking any C-terminal domain.

Similarity in domains organization was also shown in terms of evolutionary relationship after sequence comparisons via multiple sequence alignment (MSA). Such alignment resulted in 656 conserved sites, 158 variable residues over 815 amino acidic residues and in absence of parsimony informative substitutions. Moreover, MSA analysis of collagenases secreted by *V*. *alginolyticus* and *V. parahaemolyticus* showed that VppC and VPM shared identity higher than 80% with VAC collagenase from *V*. *alginolyticus*. 

**Figure 5:**
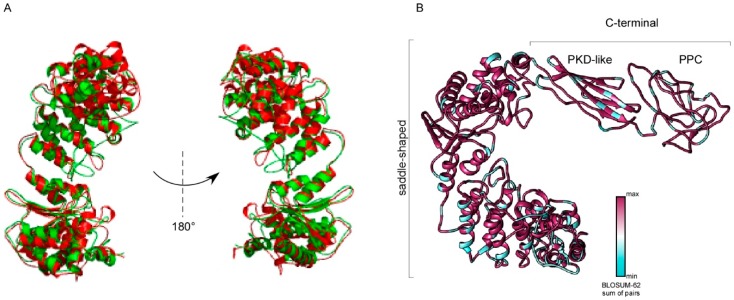
Ribbon diagrams of the collagenase structures herein defined. The 3D structures were created via the Phyre 2 software and rendered by using Chimera package. (**A**) Superposition of three-dimensional models in ribbon representation of the catalytic domain of VppC (green) and VAC (red). (**B**) Three-dimensional model of VppC with corresponding structure colored according to the conservation of amino acids among class III collagenase of M9A subfamily from *V. alginolyticus* and *V. parahaemolyticus*. The 3D structures were created via the Phyre 2 software and rendered by using Chimera package. Variable positions are presented in light-blue; while conserved amino acids are showed in magenta as defined in the color-coding bar.

An effort was also made to identify similarities of protein folds. Toward this end, the 3D structure of these collagenase was predicted by homology modelling (Figure 5A) and the results highlight the typical saddle shape configuration with a two-domain structure organized in the collagenase catalytic domain and the accessory domain containing PKD-like and PPC motifs of these enzyme. Moreover, conservation analysis among members of the collagenase class III family from *V*. *alginolyticus* and *V. parahaemolyticus* confirmed the high degree of conservation among these enzymes (Figure 5B).

In light of the results obtained from experiments with the two *Vibrio* strains, including the high responsivity of *V. parahaemolyticus* and its enzyme production performances, which greatly exceeded those of *V. alginolyticus* in many conditions, as well as the high similarity of the considered collagenases, as highlighted by the structure computational comparison, we wonder if the use of *V. parahaemolyticus* collagenase could be conceivable, instead of that from *V. alginolyticus*. 

### 3.4. Other Secreted Proteases

Given the various potential applications of proteolytic enzymes, ranging from wastes processing to biomedical/pharmaceutical uses [82], the overall exo-protease production was assessed and measured as caseinolytic activity in supernatants from each culture.

The proteases production showed differentiated responses to the various conditions the two strains were challenged with.

**Figure 6:**
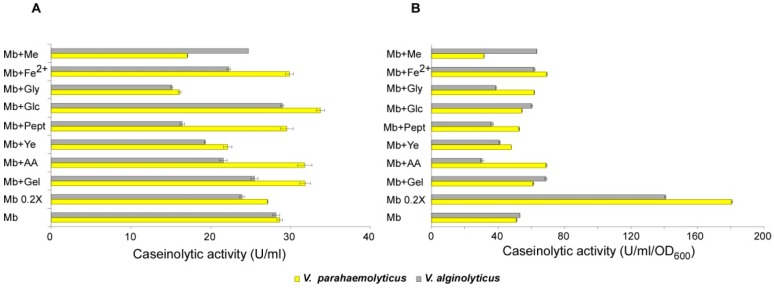
Proteases production from *V. parahaemolyticus* and *V. alginolyticus* in different culture media. The caseinolytic activity was measured using a conjugate, chromogenic substrate, incubated with supernatants from liquid cultures of *V. parahaemolyticus* and *V. alginolyticus*, grown overnight in the specified liquid media. (**A**) Caseinolytic activity measured by colorimetric test. (**B**) Caseinolytic activity normalized with cell density.

In *V. alginolyticus*, all tested supplementations except glucose (which did not induce detectable responses) resulted in reduced proteases production (Figure 6A). The strongest variations were observed for glycerol as well as for all hydrolysates but meat extract. Overall, these results supported the hypothesis that nutrient concentration is highly supportive for the growth (see Figure 1), but their availability results in repressive effects on proteases production. 

Such a concept was further confirmed and strengthened by induction data (production per cell) (Figure 6B), which highlighted that the maximum repressive effect is achieved by casaminoacids (the easiest to be uptaken) supplementation, followed by peptone and yeast extract, whereas the addition of meat extract resulted in some induction (as observed with iron, as well), probably because of the “host mimicking effect”. 

Noteworthy, the maximum proteases induction was achieved in MB 0.2×, where nutrients were five folds diluted compared with the standard medium, and where they are expected to be consumed in the early stages of the growth (note that the lowest overall growth was achieved in this medium (Figure 1)). As this means that cells grew in nutrient-limiting conditions, and then starved for a long time while producing comparable enzymes amount than in MB, it might be argued that starvation strongly stimulates enzymes production. Accordingly, nutrients availability exerts opposite effects, instead.

Similarly to what was observed for collagenase, *V. parahaemolyticus* showed a different behaviour in response to various conditions. 

As for the overall proteases production, a sharp reduction was observed only in presence of yeast extract and glycerol, whereas negligible variations or a slight increase were obtained in other media (Figure 6A). As for production per cell, meat extract resulted in repression, while little effects were recorded in all other media except for diluted MB, where the induction was much stronger than that observed in *V. alginolyticus* (Figure 6B). 

In order to get more comprehensive information about the pattern of secreted proteases in both *V. alginolyticus* and *V. parahaemolyticus*, as well as about the overall presence of metalloproteases, zimographic analyses were carried out. Gelatine was used as substrate so as to detect proteases, including collagenases, as well.

**Figure 7:**
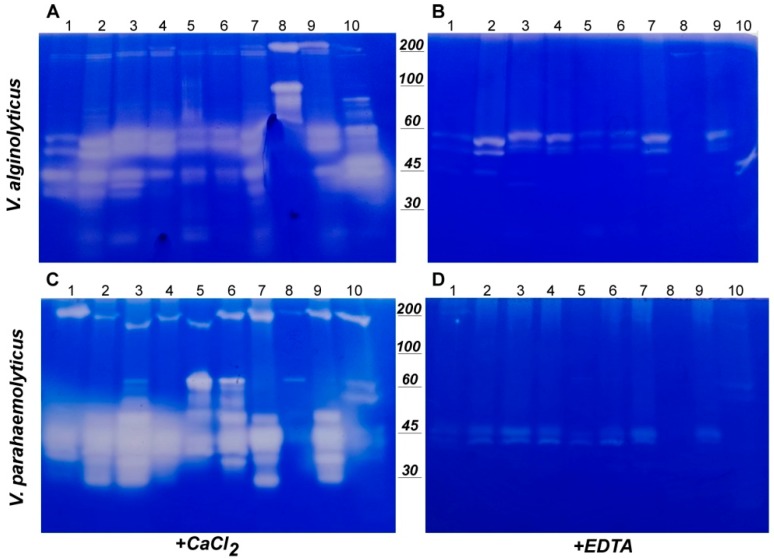
Zymographic analysis of gelatinase activity of secreted proteases. The gelatinolytic activity of supernatants from liquid cultures of *V. alginolyticus* (**A**,**B**) and *V. parahaemolyticus* (**C**,**D**) and was visualized by zymography, using gelatine as substrate. Gelatinolytic activity was measured either in presence of CaCl_2_ (**A**,**C**) or EDTA (**B**,**D**). Culture media were as follows: MB 0.2× (1); MB (2); MB+Gel (3); MB+AA (4); MB+YE (5); MB+Pept (6); MB+Glc (7); MB+Gly (8); MB+Fe (9); MB+ME (10).

*V. alginolyticus* showed nearly the same proteases patterns in all media tested, with a few additional minor bands, corresponding to low molecular weight enzymes in presence of MB 0.2×, gelatine, glucose, yeast extract and meat extract, respectively (Figure 7A).

It is noteworthy that the only exception was observed in presence of glycerol, which resulted in a severely altered pattern, where all bands observed in other conditions disappeared, being replaced by high molecular weight ones, the highest of which yielded signals at about 100 and 200 KDa, respectively. 

Interestingly, some faint digestion signals corresponding to about 200 KDa enzymes were observed in all samples; however, the signal corresponding to the highest and more intense one observed in presence of glycerol was increased in presence of iron. Similarly, casaminoacids and glucose supplementation resulted, although at much lesser extent, in the appearance of the same bands. 

Secreted proteases harbouring such apparent molecular weights have never been observed or reported in such vibrios and, to our knowledge, genomic sequences lack ORF encoding such long proteases, so that they might be worthy of further investigations, even if we cannot exclude that they could be aggregates (as the fractionation was carried out in non-reducing conditions).

A more variegated scenario was observed in *V. parahaemolyticus*, the gelatinolytic pattern of which showed more evident differences between various media (Figure 7C). In particular, the addition of gelatine, as well as of yeast extracts, peptone and meat extract, resulted in a more complex pattern, characterized by the presence of bands in the range above 60 KDa.

In *V. parahaemolyticus,* the growth in presence of glycerol resulted in much more severe effects, as any proteolytic activity nearly disappeared, and only a single, very faint band (of about 70 KDa) was still clearly visible in the pattern below 100 KDa.

A similar reduction was observed in the highest molecular weight band, of about 220 KDa. In fact, in *V. parahaemolyticus* digestion bands (only one per lane), different among various conditions, were clearly visible in the range of about 170–220 KDa. Their presence, similarly to what discussed for *V. alginolyticus*, was unexpected and not previously reported, so that further investigations would be needed in order to better characterize them.

Inhibition tests in presence of EDTA (Figure 7B,D) showed that most of the bands observed in both strains, including the highest ones, correspond to metalloproteases.

## 4. Conclusions

Overall, data reported herein highlight the possibility of improving the enzymes production in autologous systems, although in most cases recombinant production offers undisputed advantages, including the possibility of improved cloning and expression in standard microbial factories like *E. coli* [83], as well as the production from toxin-free organisms through improved expression systems [82].

A modified medium, for improved collagenases production in *V. parahaemolyticus*, was reported, where the combined supplementation of two non-animal inducers (namely, iron and glucose) has proven to be beneficial. Similarly, starvation (i.e., growth in low nutrient concentration medium) was shown to enhance proteases production in *V. alginolyticus.*

Moreover, the possibility of expanding the repertoire of available collagenases for biomedical applications is proposed, supported by bioinformatics data and technological considerations, as well.

Furthermore, the results elicited intriguing insights into regulatory mechanisms, worthy of further studies involving multiple levels, ranging from transcriptional to post-transcriptional regulation, including mRNAs metabolism, as well as protein synthesis and secretion.
